# Insights Into Tertiary Care Nurses: Awareness and Practices Regarding Nonpharmacological Pediatric Pain Management in Qatar

**DOI:** 10.1002/pne2.70018

**Published:** 2025-12-09

**Authors:** Jibin Kunjavara, Rajesh Rai, Kalpana Singh, Abdulqadir Nashwan, Badriya Lenjawi

**Affiliations:** ^1^ Nursing and Midwifery Research Department Hamad Medical Corporation Doha Qatar; ^2^ Dy. Patil University Navi Mumbai India; ^3^ University of Doha for Science and Technology Doha Qatar

**Keywords:** knowledge, non‐pharmacological pain management, pediatric nursing, practices, Qatar, tertiary hospitals

## Abstract

Effective pain management, particularly non‐pharmacological pain management (NPPM), is a critical component of pediatric care. Tertiary care settings play a pivotal role in implementing and modeling best practices in NPPM due to their specialized services, multidisciplinary teams, and access to advanced resources. As tertiary public hospitals often handle complex and severe pediatric cases, they serve as a benchmark for high‐quality, holistic pain management practices, including non‐pharmacological approaches. This study aimed to assess the knowledge, attitudes, and perceived practices of pediatric nurses regarding NPPM in tertiary public hospitals in Qatar. Additionally, the study explored the association between nurses' demographic characteristics and their competencies in NPPM. A cross‐sectional design was employed. Data were collected from 136 pediatric nurses in emergency and inpatient units of tertiary public hospitals between August and October 2024. Proportionate sampling was used to select participants. A structured, self‐administered questionnaire adapted from validated tools assessed knowledge, attitudes, and practices. Ethical approval was obtained, and data collection adhered to privacy and confidentiality protocols. The mean age of participants was 36.9 ± 5.7 years, with most reporting workload challenges (94.1%) and insufficient NPPM resources (58.8%). The nurse‐to‐patient ratio was most reported as 1–4 (58.1%), with a smaller proportion working under undetermined ratios (39.0%). A majority (94.1%) reported experiencing workload challenges, and 58.8% reported insufficient NPPM resources. The mean knowledge score was 10.4 ± 2.1, reflecting moderate understanding. While 86.8% correctly identified the best judge of a patient's pain intensity, gaps in knowledge regarding chronic pain management and pediatric pain assessment were evident. The mean attitude score was 50.3 ± 5.9, indicating a positive outlook toward NPPM, though only 41.9% deemed their training adequate. Practices revealed a mean score of 57.3 ± 6.8, with high usage of preparation techniques (87.5%) and verbal reassurance (86.7%). However, methods like guided imagery were underutilized (47.8%). Family involvement in pain management was emphasized, with 92.6% of nurses integrating family participation. This study found that pediatric nurses demonstrate positive attitudes toward non‐pharmacological pain management (NPPM) but face gaps in chronic pain management and pediatric pain assessment. Nurse‐to‐patient ratios also influenced practice, with heavier workloads limiting individualized care. Targeted education, interprofessional collaboration, adequate resources, and evidence‐based staffing are essential to strengthen NPPM competencies and improve pediatric pain outcomes. Despite positive attitudes and the adoption of certain effective practices, significant gaps remain in knowledge and the consistent application of NPPM techniques among pediatric nurses in tertiary care settings.

## 1 Background

Pain, recognized by the World Health Organization (WHO) as the fifth vital sign [1], is a common and distressing experience in intensive care units (ICUs) [2]. Critically ill patients frequently endure moderate to severe pain, both at rest and during routine care such as suctioning or wound dressing. Acute pain is a short‐term, protective response to injury or disease, associated with muscle spasm and sympathetic activation, whereas chronic pain persists beyond normal healing, often lacking biological purpose and potentially influenced by psychological factors [3]. Pediatric pain also imposes a substantial economic burden, with costs in the United States estimated at $19.5 billion annually [4].

The majority of patients living with pain rely on opioids for relief. However, prolonged use of these medications can have adverse effects, including dependence, impaired memory, drowsiness, tolerance, reduced concentration, and poor judgment. Nonpharmacological methods for managing children's pain can be categorized into sensory or physical interventions, cognitive approaches, and cognitive‐behavioral techniques. Sensory interventions focus on stimulating specific body areas or applying peripheral skin stimulation to alleviate pain [5].

Non‐pharmacological pain management (NPPM) strategies are tailored to developmental stages to ensure age‐appropriate care. Infants benefit from physical and emotional comfort measures such as swaddling, non‐nutritive sucking, sucrose, clustering procedures, and calming with music or a familiar face. Toddlers and preschoolers respond well to parental presence, procedural preparation, positive reinforcement, distraction, therapeutic play, and activities like blowing bubbles. School‐age children benefit from cognitive and physical approaches, including guided imagery, distraction, deep breathing, heat or cold therapy, and therapeutic art or play [6]. Adolescents respond to procedural preparation, guided imagery, relaxation techniques, privacy adjustments, and spiritual meditation, supporting emotional security and effective coping [7]. Cognitive strategies, which redirect attention from pain, are more suitable for older children with symbolic thinking and verbal skills, while cognitive‐behavioral interventions combine mental and physical activities to enhance central pain control, reduce anxiety, and improve adaptive coping alongside medications for moderate to severe pain [8].

Nonpharmacological pain management (NPPM) strategies offer several advantages over pharmacological methods, including cost‐effectiveness, ease of implementation, high potential for pain relief, and the ability to be used alone or in combination with medications [9]. Studies have shown that nurses can enhance their knowledge of NPPM through work experience, on‐the‐job training, and peer interactions [10]. Research conducted in Turkey indicated that emotional support methods were the most frequently employed, while others highlighted the use of cognitive‐behavioral strategies. Pediatric nurses in Turkey often feel competent in providing emotional support, possibly influenced by cultural and social factors [11].

Pediatric nurses utilize various non‐pharmacological pain management (NPPM) techniques, including preoperative education, reassurance, creating a comfortable environment, distraction, positioning, emotional support, and assistance with daily activities [12]. Studies from Egypt and other settings highlight interventions such as massage therapy for infants and the use of cartoons or informational videos during procedures, which reduce pain and fear while improving nursing care quality [13, 14]. Globally, nurses' limited knowledge and unfavorable attitudes remain significant barriers to NPPM implementation [15]. Research, including studies from Iran, shows that adequate knowledge and positive attitudes are critical for effective pain management, as nurses lacking these often struggle to apply NPPM strategies [16]. Despite its proven benefits, NPPM adoption in Qatar is limited, underscoring the need to explore pediatric nurses' knowledge, attitudes, and practices in the local context.

## 2 Research Questions

- What is the level of knowledge, attitudes, and commonly practiced non‐pharmacological pain management techniques among pediatric nurses in clinical settings in Qatar?
- Is there an association between pediatric nurses' demographic characteristics (e.g., age, years of experience, educational background) and their knowledge, attitudes, and practices related to NPPM?
- What are the perceived barriers and facilitators to implementing non‐pharmacological pain management strategies in pediatric care settings in Qatar?

## 3 Methods

### 3.1 Study Design

This study used initial data from a quasi‐experimental design to look at the characteristics before the intervention and to explore the connections between knowledge, attitude, and practice (KAP) levels and demographic factors. The study concentrates on pre‐intervention data to investigate the connections between KAP scores and the demographic characteristics of the participants. Data were collected between August and October 2024 across tertiary public hospitals.

### 3.2 Study Setting

The study was conducted at tertiary hospitals representing the public healthcare system of Qatar. The pediatric emergency and inpatient departments of Hamad Medical Corporation (HMC) were the selected areas for the study. These facilities employ a diverse nursing workforce, ranging from clinical nurses delivering bedside care to executive‐level nursing administrators. The study was conducted across seven pediatric care facilities in Qatar, which included a total nursing population of 456. Using the proportionate sampling method, 30% of nurses from each facility were selected through simple random sampling from staff lists, resulting in a sample size of 136, all of whom were female, as there were no male nurses in the population. Participants were drawn from the Pediatric Ward and PICU at Hamad General Hospital, Pediatric Emergency Centers under the Al Sadd zone (including Al Daayen, Al Rayyan, and Airport), and the Emergency Department at Al Wakrah Hospital. This sampling approach ensured balance and representative participation from each clinical setting.

### 3.3 Inclusion and Exclusion Criteria

#### 3.3.1 Inclusion Criteria

The inclusion criteria include registered nurses who hold a valid nursing license. Nurses working in the pediatric department, including staff nurses, charge nurses, head nurses, nurse specialists, and nurse educators, must have at least 1 year of experience in their current organization.

#### 3.3.2 Exclusion Criteria

Nurses on extended leave or unavailable during the data collection period were excluded from the study.

### 3.4 Sampling and Sample Size

The two pediatric nursing emergency departments and three inpatient units, including the PICU, employed over 456 nurses from various nationalities, which contributed to a diversity of experience and knowledge levels. The sample was selected using the proportionate sampling technique, which involved taking 30% of the sample from each facility based on the population distribution requirements. The calculated sample size was 122, Innab et al. [17] and to account for a 10% attrition or non‐response rate, it was increased to 136. This calculation was based on a 5% margin of error and a 95% level of significance.

### 3.5 Description of the Tool

The data collection tool used in this study was a structured, self‐administered questionnaire (NKASRP), adapted from similar studies [18, 19, 20]. The questionnaire was in English and consisted of closed‐ended questions. The first section included 14 sociodemographic questions regarding the participants' age, gender, educational status, years of experience, and formal training in non‐pharmacological pain management. The second section comprised 16 questions to assess the participants' knowledge, with responses scored as ‘1’ for correct answers and ‘0’ for incorrect answers. The third section consisted of 17 questions employing a Likert scale to assess the participants' attitudes toward non‐pharmacological pain management strategies. The scoring system used for these questions was SD (Strongly Disagree), D (Disagree), N (Neutral), A (Agree), and SA (Strongly Agree). The fourth section, adopted from Bicek [18], aimed to assess the nurses' self‐reported practices regarding the use of non‐pharmacological pain management strategies. It included six domains: preparing the patient for a procedure (2 questions), imagery (4 questions), distraction (3 questions), relaxation techniques (2 questions), physical methods (3 questions), and emotional support (1 question). The practice section was scored on a five‐point scale: 1 = Not at all, 2 = Very seldom, 3 = Sometimes, 4 = Nearly always, and 5 = Always. Permission was obtained from the authors to use the scales via email, and all scales were tested for reliability with a 0.82 score, and content validity was assessed.

#### 3.5.1 Reliability and Validity of the Tool

The content validity of the study tool was ensured through a structured review process involving six experts from diverse pediatric healthcare and academic backgrounds. The panel included two Clinical Nurse Specialists (CNSs), one specializing in Pain Management and the other in Pediatrics, who assessed the tool's alignment with best practices in pediatric pain management. Additionally, two Pediatric Nurse Educators evaluated the tool's clarity, educational relevance, and its suitability for assessing nurses' knowledge, attitude, and practice (KAP). The internal consistency of the Knowledge, Attitude, and Practices (KAP) scales was assessed using Cronbach's Alpha, both before and after the intervention. The Knowledge scale showed excellent reliability, with a pretest alpha of 0.875 indicating consistently high internal consistency across the 16 items. The Attitude scale demonstrated acceptable reliability at the pretest (*α* = 0.758). Similarly, the Practices scale exhibited acceptable internal consistency before the intervention (*α* = 0.752).

### 3.6 Recruitment and Data Collection

We collected data through a face‐to‐face interaction using a paper‐printed questionnaire. The survey evaluated eligibility, knowledge, attitudes, and practices related to NPPM among pediatric nurses. To analyze potential associations with the scores.

After receiving approval from the medical research center, the principal investigator collaborated with the director of nursing and head nurses from the pediatric emergency and inpatient units to compile a list of potential participants. We then invited the prospective study subjects to participate via their corporate emails, along with an information sheet. We contacted those willing to participate through email.

The study took place over the course of 6 months, from August 2024 to October 2025. The principal investigator and research team organized an informative session at the hospitals to meet potential participants and explain the study's objectives and procedures. The research team invited interested individuals to participate, provided opportunities for questions, and outlined the consent process. On the scheduled day for data collection, the research team distributed questionnaires to participants who agreed to take part. The participants were allotted 15–20 min to complete the questionnaires, after which the completed forms were collected. To protect the participants' privacy and ensure the security of their information, each participant was assigned a unique code, which was used instead of personal identifiers throughout the data collection and analysis process.

### 3.7 Ethical Considerations

The study received ethical approval from the Institutional Review Board (IRB) of the healthcare organization under the Medical Research Center (Protocol No. MRC‐01‐23‐441). We informed the participants about the voluntary nature of the study and assured them of their anonymity. Completion of the survey implied informed consent, and the privacy and confidentiality of the data were assured.

### 3.8 Statistical Analysis Plan

Descriptive statistics were used to summarize the variables, presenting categorical data such as demographic characteristics and knowledge, attitude, and practice (KAP) levels as frequencies and percentages, while continuous variables were summarized using means and standard deviations. Independent t‐tests were conducted to assess associations between KAP scores and demographic factors with two categories, while one‐way ANOVA was used for variables with more than two categories. STATA 17.0 software was used to analyze the data. A *p*‐value of < 0.05 was considered statistically significant for all analyses.

## 4 Results

Data normality refers to the extent to which the distribution of data follows a normal (bell‐shaped) curve. Assessing normality is essential because many statistical tests, such as *t*‐tests and ANOVA, assume that the data are normally distributed. Visual methods such as histograms and Q‐Q plots, along with statistical tests like the Shapiro–Wilk or Kolmogorov–Smirnov tests, can evaluate normality. If the data are normally distributed, parametric tests can be used; if not, non‐parametric alternatives are recommended [21].

### 4.1 Demographic Data

Table 1 depicts the 136 participants, all of whom were female nurses working in pediatric inpatient units. The mean age of participants was 36.9 ± 5.7 years. The most commonly reported nurse‐to‐patient ratio was 1–4 (58.1%), while a smaller proportion of participants worked under undetermined ratios (39.0%). A majority (94.1%) reported experiencing workload, and 58.8% indicated the absence of NPPM (non‐pharmacological pain management) equipment, while 52.9% stated that NPPM guidelines were not available in their units.

**TABLE 1:** Participant characteristics.

Variables	Response	Value
*N*		136
Nurse to patient ratio	Undetermined	53 (39.0%)
	1to 4	79 (58.1%)
	1 to 6	1 (0.7%)
	1to 8	3 (2.2%)
Presence of workload (challenges)	No	8 (5.9%)
	Yes	128 (94.1%)
Is there any NPPM equipment	No	80 (58.8%)
	Yes	56 (41.2%)
Availability of NPPM guideline	No	72 (52.9%)
	Yes	64 (47.1%)
Working hours	8 h/day	35 (25.7%)
	9–12 h/day	58 (42.6%)
	> 12 h/day	43 (31.6%)
Availability of pain assessment tool	No	4 (2.9%)
	Yes	132 (97.1%)
Do you have training on NPPM	No	100 (73.5%)
	Yes	36 (26.5%)
Previous education on NPPM	No	67 (49.3%)
	Yes	69 (50.7%)
Have you ever used NPPM	No	32 (23.5%)
	Yes	104 (76.5%)
Working unit	Inpatient	82 (60.2%)
	Emergency	54 (39.8%)
Age, mean (SD)		36.9 (5.7)
Sex	Female	136 (100.0%)
Educational status	Diploma	10 (7.4%)
	Bachelor's degree	115 (84.6%)
	Master's degree	11 (8.1%)
Experience	1–5 year	9 (6.6%)
	5–10 year	59 (43.4%)
	10 years above	68 (50.0%)

*Note:* Mean (SD) scores represent participants' levels of knowledge, attitude, and practice regarding NPPM. *p*‐values indicate the results of statistical comparisons (e.g., independent *t*‐tests or one‐way ANOVA) between groups for each variable.

Abbreviations: *N*, number of participants in each category; NPPM, non‐pharmacological pain management; SD, standard deviation.

Regarding working hours, 42.6% of nurses worked between 9 and 12 h/day, followed by 31.6% who worked more than 12/day hours, and 25.7% who worked 8‐h shifts/day. Most nurses (97.1%) reported the availability of pain assessment tools, though only 26.5% had received training on NPPM, and 50.7% had previous education on the subject. Additionally, 76.5% used NPPM in their practice. In terms of educational status, 84.6% had a bachelor's degree, 8.1% had a master's, and 7.4% had a diploma. Half of the nurses had over 10 years of experience, while 43.4% had 5–10 years, and 6.6% had 1–5 years of experience.

The study looked at what nurses knew about pain management and non‐drug methods for managing pain, with an average knowledge score of 10.4. Most nurses (86.8%) correctly identified the most accurate judgment of a patient's pain intensity, and 94.1% demonstrated an understanding of factors that alleviate pain. However, only 27.2% correctly identified approaches for alleviating chronic pain. Similarly, 89.7% understood the role of involvement in improving pain management, while 67.6% recognized effective methods for managing children's pain. About 47.8% correctly identified ways to reduce pain sensation, and only 22.8% knew the most reliable indicator for assessing children's pain.

When addressing mild pain, 66.9% provided correct responses, and 62.5% were aware that modeling desired behaviors was not applicable for infants in NPPM. Regarding the benefits of NPPM, 66.9% answered correctly, while 96.3% knew how to decrease the perception of pain. A significant majority (80.1%) understood the importance of proper pain management for children with chronic pain, and 66.2% identified acceptable NPPM methods for preschool‐aged children. However, only 39.7% correctly answered what actions could not help calm children. Moreover, 70.6% identified physical interventions for NPPM, and 57.4% recognized recommended NPPM methods for toddlers.

For attitudes, the mean score was 50.3 ± 5.9, with 43.4% classified as having low attitudes, 29.4% moderate, and 27.2% high attitudes. The study evaluated nurses' attitudes toward various aspects of non‐pharmacological pain management (NPPM) with a mean attitude score of 50.3 ± 5.9 (Table S3). Most nurses (74.3%) disagreed with or strongly disagreed with the statement that pain cannot be seen in patients' behavior, and 66.9% believed they were willing to provide NPPM methods to patients. Regarding the adequacy of NPPM education during training, 41.9% agreed it was sufficient, while 37.5% disagreed. A majority (58.1%) were willing to provide information related to NPPM methods to parents, and 70.6% felt that encouraging children to focus on positive matters could relieve pain (Table S2).

Opinions were divided on whether preparing a patient for a procedure could decrease pain, with 54.4% agreeing or strongly agreeing, and on the use of both pharmacological and non‐pharmacological methods together, where 67% disagreed or firmly disagreed with the statement that it was inadvisable. Additionally, 60.3% disagreed that teaching breathing techniques cannot relieve pain, while 60.3% also agreed or strongly agreed that encouraging relaxation could alleviate it. A notable 76.5% were willing to encourage family members to bring children's belongings to the hospital to enhance comfort (Table S3).

The study evaluated nurses' practices in non‐pharmacological pain management (NPPM) with a mean practice score of 57.3 ± 6.8. Most nurses (87.5%) consistently prepared patients for procedures by explaining them, with 52.2% always doing so. Similarly, a significant proportion (66.1%) encouraged children to imagine pleasant and positive matters during painful experiences, with 25.7% always practicing this. Encouraging relaxation of body parts (67.6%) and teaching correct breathing techniques (69.9%) were frequently used methods to alleviate pain. Verbal reassurance was commonly practiced, with 86.7% of respondents indicating they used it nearly always or always (see Table S3). With 50% or more practicing them nearly always or always. Family involvement was emphasized, as 79.4% of nurses consistently encouraged family members to bring a child's belongings, and 92.6% included them in the pain management regimen. Guided imagery techniques were less commonly used, with 47.8% practicing them nearly always or always.

Table 2 illustrates that the mean knowledge scores show no significant differences across most variables except for equipment availability and guideline presence. Nurses with access to equipment had a significantly lower mean knowledge score (9.9 ± 1.9) compared to those without (10.8 ± 2.2, *p* = 0.011). Similarly, nurses with available guidelines had a slightly lower knowledge score (10.0 ± 2.0) than those without (10.8 ± 2.2, *p* = 0.033).

**TABLE 2:** Association with demographic variables with NPPM, knowledge attitude, and practice score.

Variables	*N*	Knowledge, mean (SD)	Attitude, mean (SD)	Practice, mean (SD)
Presence of workload				
No	8	11.0 (1.2)	49.9 (2.7)	58.4 (6.3)
Yes	128	10.4 (2.2)	50.3 (6.1)	57.3 (6.8)
*p*		0.43	0.83	0.66
Equipment				
No	80	10.8 (2.2)	49.6 (5.8)	56.5 (6.3)
Yes	56	9.9 (1.9)	51.3 (6.0)	58.6 (7.2)
*p*		0.011	0.091	0.080
Guideline availability				
No	72	10.8 (2.2)	49.7 (5.2)	56.4 (6.9)
Yes	64	10.0 (2.0)	51.0 (6.6)	58.4 (6.5)
*p*		0.033	0.18	0.074
Working hours				
8 h	35	10.2 (1.9)	50.1 (6.0)	57.7 (7.1)
9–12 h	58	10.5 (2.2)	51.0 (6.0)	56.5 (6.8)
> 12 h	43	10.5 (2.3)	49.6 (5.7)	58.2 (6.5)
*p*		0.81	0.45	0.42
Availability of pain assessment tool				
No	4	10.8 (1.0)	51.0 (2.7)	54.3 (5.1)
Yes	132	10.4 (2.1)	50.3 (6.0)	57.4 (6.8)
*p*		0.76	0.82	0.36
Training on NPPM				
No	100	10.5 (2.1)	50.4 (5.5)	57.3 (6.7)
Yes	36	10.1 (2.1)	50.1 (7.1)	57.3 (6.9)
*p*		0.30	0.81	0.97
Previous education on NPPM				
No	67	10.4 (1.9)	49.5 (6.2)	57.3 (7.0)
Yes	69	10.4 (2.3)	51.1 (5.5)	57.3 (6.6)
*p*		0.91	0.12	0.99
Used NPPM				
No	32	10.3 (2.3)	49.9 (5.2)	56.3 (7.9)
Yes	104	10.5 (2.1)	50.4 (6.1)	57.7 (6.4)
*p*		0.80	0.66	0.31
Nurse to patient ratio				
Undetermined	53	10.3 (1.7)	50.2 (5.8)	59.1 (6.5)
1 to4 ratio	79	10.5 (2.4)	50.1 (5.8)	56.0 (6.5)
1 to 6 or8 ratio	4	10.3 (1.7)	56.8 (6.4)	60.3 (10.9)
*p*		0.84	0.086	0.021

*Note:* Mean (SD) scores represent participants' levels of knowledge, attitude, and practice regarding NPPM. *p*‐values indicate the results of statistical comparisons (e.g., *t*‐tests or ANOVA) between groups for each variable.

Abbreviations: *N*, number of participants in each category; NPPM, non‐pharmacological pain management; SD, standard deviation.

The mean practice scores showed a significant difference based on the nurse‐to‐patient ratio, where nurses in the 1:6 or 1:8 ratio category had the highest practice score (60.3 ± 10.9) compared to those with undetermined or 1:4 ratios (*p* = 0.021). Although nurses with access to equipment and guidelines had slightly higher practice scores compared to those without, the differences were not statistically significant (*p* = 0.080 and *p* = 0.074, respectively).

## 5 Discussion

This study assessed pediatric nurses' knowledge, attitudes, and practices (KAP) regarding non‐pharmacological pain management (NPPM). While overall competence was moderate to high, important gaps were noted in chronic pain management and pediatric pain assessment, reflecting similar challenges reported internationally.

### 5.1 Knowledge of NPPM

Most nurses demonstrated strong foundational knowledge, with 86.8% identifying the patient as the best judge of pain and 94.1% recognizing common relieving factors, consistent with earlier findings [22]. However, specialized knowledge was limited; only 27.2% identified strategies for chronic pain, and 22.8% recognized reliable indicators for pediatric pain. Comparable deficits have been reported in Norway and Ethiopia, where 40%–60% of nurses lacked effective use of validated tools such as FLACC and Wong‐Baker FACES [23, 24], and in Saudi Arabia [25]. Interestingly, nurses with access to equipment and guidelines had lower mean knowledge scores, suggesting reliance on protocols rather than personal expertise, as also noted by El‐husseiny et al. [26].

### 5.2 Attitudes Toward NPPM

Nurses expressed largely positive attitudes, with 79.4% willing to implement NPPM and 80.1% emphasizing family education. However, only 54.4% agreed that pre‐procedural preparation reduces pain, reflecting uncertainty in this area. More favorable attitudes among those with access to resources align with findings that structured tools improve confidence and clarity [27].

### 5.3 Practices of NPPM

Practice results were encouraging, with 87.5% consistently preparing patients for procedures and frequent use of relaxation, breathing, and distraction methods, supporting prior evidence [28]. Less frequent use of guided imagery (47.8%) and physical interventions may stem from limited training, awareness, or time constraints, consistent with Ko et al. [29]. Nurse‐to‐patient ratios also influenced practice: nurses caring for six to eight patients reported higher scores, possibly due to reliance on quick, accessible methods. This supports earlier findings that high ratios negatively affect individualized pain care [30].

The knowledge gaps in chronic pain and assessment mirror global reports [23, 24, 25]. These challenges highlight the need for structured and interactive training approaches, such as workshops, simulations, and case‐based learning, which have been shown to improve competencies, confidence, and outcomes [31]. While resources such as guidelines are valuable, they should complement rather than replace education to ensure sustainable improvements in pediatric pain management.

### 5.4 Limitations

While this study provides valuable insights into nurses' knowledge, attitudes, and practices of NPPM, several limitations should be acknowledged. First, data were collected through self‐reported questionnaires, which may introduce response bias and overestimate actual practice. Second, the study included only female nurses working in pediatric inpatient units, which may limit the generalizability of the findings. Future research should include a more diverse sample, incorporating male nurses and participants from varied healthcare settings, to provide a broader understanding of NPPM across different contexts.

### 5.5 Implications for Practice

Nonetheless, the study highlights several critical areas for improvement in the implementation of non‐pharmacological pain management (NPPM). First, targeted education and awareness are essential, especially in under‐addressed areas like chronic pain management and pediatric pain assessment. Integrating comprehensive NPPM training into both pre‐service curricula and ongoing professional development can help bridge knowledge gaps. In addition to education, implementing clinical mentorship programs and peer support systems may promote the consistent application of NPPM techniques. Interprofessional collaboration, especially with physiotherapists, psychologists, and pain specialists, can enhance the multidisciplinary approach to pain care. Regular clinical audits and feedback mechanisms can also encourage adherence to NPPM protocols and identify areas needing improvement. Moreover, ensuring the availability of necessary resources, such as age‐appropriate distraction tools, physical therapy aids, and standardized guidelines, is vital for effective practice. Finally, optimizing nurse‐to‐patient ratios through evidence‐based staffing policies can allow for more individualized, time‐intensive interventions that NPPM often requires in pediatric settings.

## 6 Conclusion

In conclusion, the study highlights that while pediatric nurses generally demonstrate positive attitudes and moderate levels of knowledge and practice regarding non‐pharmacological pain management (NPPM), there remain evident areas for enhancement. Targeted educational programs, improved access to resources, and the availability of clinical guidelines could significantly strengthen nurses' ability to deliver effective NPPM. Additionally, optimizing nurse‐to‐patient ratios and encouraging active family involvement in pain management may further improve outcomes for pediatric patients. Ongoing research and continuous professional development are vital to ensure pediatric nurses are well‐equipped with the necessary knowledge, skills, and tools to provide holistic and effective pain management for children.

## Funding

This work was supported by Qatar National Library.

## Conflicts of Interest

The authors declare no conflicts of interest.

## Supporting information


**Table S1:** Knowledge at baseline.
**Table S2:** Attitude at baseline.
**Table S3:** Practice at baseline.

## Data Availability

The data that support the findings of this study are available on request from the corresponding author. The data are not publicly available due to privacy or ethical restrictions.
