# Supplementary material for: Insights Into Tertiary Care Nurses: Awareness and Practices Regarding Nonpharmacological Pediatric Pain Management in Qatar
Source: Paediatr Neonatal Pain. 2025 Dec 9;7(4):e70018. doi: 10.1002/pne2.70018 (PMC12687297; doi:10.1002/pne2.70018)
Supplement: Supplementary file 1 — Table S1: Knowledge at baseline. Table S2: Attitude at baseline. Table S3: Practice at baseline. [file PNE2-7-e70018-s001.docx]

**Supplementary Table 1- Knowledge at Baseline.**

| Variables | N (%) |
| --- | --- |
| N | 136 |
| 1)The most accurate judge of the intensity of the patient’s pain is …. | 118 (86.8%) |
| 2)Factors to reduce or alleviate pain …... | 128 (94.1%) |
| 3)Chronic pain can be alleviated by providing…… | 37 (27.2%) |
| 4)Pain management can be improved by involving | 122 (89.7%) |
| 5)Pain management of the children can be effective by the……. | 92 (67.6%) |
| 6)Sensation of Pain can be reduced by………. | 65 (47.8%) |
| 7)Most reliable indicator of the pain of the children can be assessed by………. | 31 (22.8%) |
| 8)Mild pain can be easily managed by ……… | 91 (66.9%) |
| 9)Modeling desired behaviors, the method of NPPM is not used for infant……… | 85 (62.5%) |
| 10)The benefit of non-pharmacological Pain Management is……... | 91 (66.9%) |
| 11)Decrease the Perception of the pain by……. | 131 (96.3%) |
| 12)Child with chronic pain should receive the pain management with………… | 109 (80.1%) |
| 13)The acceptable method of nonpharmacological pain management for Pre-school and children……… | 90 (66.2%) |
| 14) 3-year-old boy presents to ED triage with a history of running into the edge of a glass coffee table and hitting his head. He has a laceration on his forehead that is bleeding. His mother is trying to calm him and apply pressure to his wound. The child begins screaming and thrashing when he sees the nurse and doctor. What cannot be done to help calm these children? | 54 (39.7%) |
| 15)Physical intervention for NPPM is involve…... | 96 (70.6%) |
| 16)The method recommended for the NPPM for toddler is….... | 78 (57.4%) |
| knowledge, mean (SD) | 10.4 (2.1) |

*N = Number of participants.*

*SD = Standard Deviation.*

*NPPM = Non-Pharmacological Pain Management.*

*Each item represents a knowledge-based question regarding pediatric pain and non-pharmacological pain management strategies. Values in N (%) indicate the number and percentage of participants who responded correctly to each item.*

*The mean (SD) score reflects the overall knowledge level of participants on NPPM.*

**Supplementary Table-2 Attitude at baseline.**

| Factor | Response | Value |
| --- | --- | --- |
| N |  | 136 |
| 1)Pain cannot be seen in the Patients behavior | Strongly disagree | 50 (36.8%) |
|  | Disagree | 51 (37.5%) |
|  | Neutral | 21 (15.4%) |
|  | Agree | 11 (8.1%) |
|  | Strongly agree | 3 (2.2%) |
| 2)NPPM should be given to sick Child | Strongly disagree | 25 (18.4%) |
|  | Disagree | 66 (48.5%) |
|  | Neutral | 21 (15.4%) |
|  | Agree | 17 (12.5%) |
|  | Strongly agree | 7 (5.1%) |
| 3)Distraction increases intensity of the Pain | Strongly disagree | 43 (31.6%) |
|  | Disagree | 66 (48.5%) |
|  | Neutral | 16 (11.8%) |
|  | Agree | 11 (8.1%) |
| 4)Non-Drug interventions are very effective for severe pain than mild to moderate. | Strongly disagree | 42 (30.9%) |
|  | Disagree | 50 (36.8%) |
|  | Neutral | 29 (21.3%) |
|  | Agree | 8 (5.9%) |
|  | Strongly agree | 7 (5.1%) |
| 5) Nurses are best judges of the patient’s pain intensity than doctors because they spend 24 hours with the patient. | Strongly disagree | 17 (12.5%) |
|  | Disagree | 19 (14.0%) |
|  | Neutral | 18 (13.2%) |
|  | Agree | 56 (41.2%) |
|  | Strongly agree | 26 (19.1%) |
| 6)NPPM education received in training is adequate for effective pain Management. | Strongly disagree | 10 (7.4%) |
|  | Disagree | 41 (30.1%) |
|  | Neutral | 28 (20.6%) |
|  | Agree | 51 (37.5%) |
|  | Strongly agree | 6 (4.4%) |
| 7)The Nurses role during nondrug pain management is to follow the doctors order | Strongly disagree | 45 (33.1%) |
|  | Disagree | 31 (22.8%) |
|  | Neutral | 14 (10.3%) |
|  | Agree | 40 (29.4%) |
|  | Strongly agree | 6 (4.4%) |
| 8)willing to provide information related to NPPM method to Parents | Strongly disagree | 4 (2.9%) |
|  | Disagree | 6 (4.4%) |
|  | Neutral | 17 (12.5%) |
|  | Agree | 79 (58.1%) |
|  | Strongly agree | 30 (22.1%) |
| 9)willing to provide nonpharmalogicalmethods to people who have pain | Strongly disagree | 4 (2.9%) |
|  | Disagree | 12 (8.8%) |
|  | Neutral | 12 (8.8%) |
|  | Agree | 72 (52.9%) |
|  | Strongly agree | 36 (26.5%) |
| 10)It is not advised to use both pharmacological and NPPM together | Strongly disagree | 44 (32.4%) |
|  | Disagree | 47 (34.6%) |
|  | Neutral | 14 (10.3%) |
|  | Agree | 24 (17.6%) |
|  | Strongly agree | 7 (5.1%) |
| 11)Preparing a patient for a procedure by explaining the procedure can decrease pain | Strongly disagree | 15 (11.0%) |
|  | Disagree | 19 (14.0%) |
|  | Neutral | 28 (20.6%) |
|  | Agree | 53 (39.0%) |
|  | Strongly agree | 21 (15.4%) |
| 12)Encourage the child to think about pleasant and positive matters can relive pain | Strongly disagree | 6 (4.4%) |
|  | Disagree | 8 (5.9%) |
|  | Neutral | 26 (19.1%) |
|  | Agree | 78 (57.4%) |
|  | Strongly agree | 18 (13.2%) |
| 13)Teaching children about the correct breathing technique can’t relieve pain. | Strongly disagree | 22 (16.2%) |
|  | Disagree | 47 (34.6%) |
|  | Neutral | 14 (10.3%) |
|  | Agree | 42 (30.9%) |
|  | Strongly agree | 11 (8.1%) |
| 14)Encouraging the patient by rewarding verbally cannot alleviate his/her pain | Strongly disagree | 13 (9.6%) |
|  | Disagree | 49 (36.0%) |
|  | Neutral | 35 (25.7%) |
|  | Agree | 35 (25.7%) |
|  | Strongly agree | 4 (2.9%) |
| 15)encouraging the patient to relax different parts of his body can alleviate pa | Strongly disagree | 9 (6.6%) |
|  | Disagree | 14 (10.3%) |
|  | Neutral | 31 (22.8%) |
|  | Agree | 69 (50.7%) |
|  | Strongly agree | 13 (9.6%) |
| 16)Internal decoration of units does not affect the patient’s ability to manage the pain. | Strongly disagree | 10 (7.4%) |
|  | Disagree | 44 (32.4%) |
|  | Neutral | 42 (30.9%) |
|  | Agree | 35 (25.7%) |
|  | Strongly agree | 5 (3.7%) |
| 17)Are you willing to encourage family members to bring of child belongings to the unit. | Strongly disagree | 2 (1.5%) |
|  | Disagree | 5 (3.7%) |
|  | Neutral | 33 (24.3%) |
|  | Agree | 69 (50.7%) |
|  | Strongly agree | 27 (19.9%) |
| Attitude, mean (SD) |  | 50.3 (5.9) |

*N = Number of participants.SD = Standard Deviation, NPPM = Non-Pharmacological Pain Management.*

*Each item reflects participants’ attitudes toward various aspects of pediatric pain and NPPM.*

*Responses are measured using a 5-point Likert scale ranging from Strongly Disagree to Strongly Agree.*

*Values in N (%) indicate the number and percentage of participants selecting each response category.*

*The mean (SD) score represents the average attitude score across all items, indicating the overall attitude level toward NPPM.*

**Supplementary Table-3 Practice at Baseline**

| Variables | Response | Value |
| --- | --- | --- |
| N |  | 136 |
| 1)I prepare a patient carefully for procedure by telling him/her about what will be done. | very seldom | 1 (0.7%) |
|  | sometimes | 16 (11.8%) |
|  | nearly always | 48 (35.3%) |
|  | always | 71 (52.2%) |
| 2)I encourage the child to think about imagine pleasant and positive matters when she feels pain. | Not at all | 2 (1.5%) |
|  | very seldom | 11 (8.1%) |
|  | sometimes | 33 (24.3%) |
|  | nearly always | 55 (40.4%) |
|  | always | 35 (25.7%) |
| 3)which of the following things do you usually give to the child during painful procedure. | Not at all | 4 (2.9%) |
|  | very seldom | 16 (11.8%) |
|  | sometimes | 79 (58.1%) |
|  | nearly always | 32 (23.5%) |
|  | always | 5 (3.7%) |
| 4)I encourage the child to relax various parts of his body to alleviate the sensation of pain. | Not at all | 2 (1.5%) |
|  | very seldom | 6 (4.4%) |
|  | sometimes | 36 (26.5%) |
|  | nearly always | 71 (52.2%) |
|  | always | 21 (15.4%) |
| 5)I teach the elder child to correct breathing technique to alleviate pain. | Not at all | 1 (0.7%) |
|  | very seldom | 11 (8.1%) |
|  | sometimes | 29 (21.3%) |
|  | nearly always | 56 (41.2%) |
|  | always | 39 (28.7%) |
| 6)when a child has pain, I encourage the patient by rewarding he/she verbally | Not at all | 4 (2.9%) |
|  | very seldom | 14 (10.3%) |
|  | sometimes | 40 (29.4%) |
|  | nearly always | 61 (44.9%) |
|  | always | 17 (12.5%) |
| 7)I use thermal regulation as a method of pain relief | Not at all | 3 (2.2%) |
|  | very seldom | 14 (10.3%) |
|  | sometimes | 51 (37.5%) |
|  | nearly always | 52 (38.2%) |
|  | always | 16 (11.8%) |
| 8)I use desensitization techniques to relive pain | Not at all | 13 (9.6%) |
|  | very seldom | 14 (10.3%) |
|  | sometimes | 60 (44.1%) |
|  | nearly always | 42 (30.9%) |
|  | always | 7 (5.1%) |
| 9)I alleviate patients pain by position changes | Not at all | 1 (0.7%) |
|  | very seldom | 4 (2.9%) |
|  | sometimes | 28 (20.6%) |
|  | nearly always | 75 (55.1%) |
|  | always | 28 (20.6%) |
| 10)I used to apply distraction techniques to relive pain among children | very seldom | 1 (0.7%) |
|  | sometimes | 23 (16.9%) |
|  | nearly always | 64 (47.1%) |
|  | always | 48 (35.3%) |
| 11)I verbally comfort and reassure the Patient. | very seldom | 4 (2.9%) |
|  | sometimes | 14 (10.3%) |
|  | nearly always | 63 (46.3%) |
|  | always | 55 (40.4%) |
| 12)I use touching as a method of pain relief | very seldom | 8 (5.9%) |
|  | sometimes | 23 (16.9%) |
|  | nearly always | 59 (43.4%) |
|  | always | 46 (33.8%) |
| 13)I try to alleviate pain by guided imagery techniques for pain management the child | Not at all | 12 (8.8%) |
|  | very seldom | 12 (8.8%) |
|  | sometimes | 47 (34.6%) |
|  | nearly always | 44 (32.4%) |
|  | always | 21 (15.4%) |
| 14)I encourage family members to bring some of the child belongings to the unit | Not at all | 2 (1.5%) |
|  | very seldom | 4 (2.9%) |
|  | sometimes | 22 (16.2%) |
|  | nearly always | 66 (48.5%) |
|  | always | 42 (30.9%) |
| 15)I include family members in the pain management regimen | Not at all | 1 (0.7%) |
|  | sometimes | 9 (6.6%) |
|  | nearly always | 54 (39.7%) |
|  | always | 72 (52.9%) |
| Practice, mean (SD) |  | 57.3 (6.8) |

*N = Number of participants.SD = Standard Deviation.NPPM = Non-Pharmacological Pain Management. This table presents the self-reported frequency of various non-pharmacological practices used by participants in pediatric pain management. Each item was rated using a 5-point Likert scale with the following options: Not at all, very seldom, Sometimes, nearly always, and always.*
